# P-1661. COVID-19 Vaccine Uptake in Children Aged 1 - 4 Years from State Vaccine Registry Data through March 2024

**DOI:** 10.1093/ofid/ofaf695.1836

**Published:** 2026-01-11

**Authors:** Tara Ahi, Jazmine S Mateus, Tiange Yu, Anan Zhou, Rajeev M Nepal, Mary M Moran, Alejandro Cané, Santiago M C Lopez, Laura A Puzniak, Kathleen M Andersen

**Affiliations:** Pfizer Inc, New York, NY; Genesis Research Group, Hoboken, New Jersey; Genesis Research Group, Hoboken, New Jersey; Genesis Research Group, Hoboken, New Jersey; Pfizer Canada, Krikland, Quebec, Canada; Pfizer Inc., Collegeville, Pennsylvania; Pfizer Inc., Collegeville, Pennsylvania; Pfizer Inc, New York, NY; Pfizer Inc., Collegeville, Pennsylvania; Pfizer Inc., Collegeville, Pennsylvania

## Abstract

**Background:**

In the United States, COVID-19 mRNA vaccines are authorized for use in children aged 6 months to 4 years as a multidose primary series. Although this age group had the highest rate of weekly COVID-19 associated hospitalizations among pediatrics from October 2022-March 2025, there is limited data on vaccine uptake. The aim of this study was to determine the proportion of children aged 1-4 years who were compliant with CDC-recommended 2023-2024 COVID-19 vaccine dose(s).

**Methods:**

COVID-19 vaccine administration was evaluated among children born between 2019-2022 (ages 1 – 4 in 2023) living in California and Louisiana, two states with mandatory vaccine reporting. We evaluated uptake of the 2023-2024 XBB.1.5-adapted formulation between September 2023-March 2024 and their historical vaccine receipt such as receipt of wildtype or 2022-2023 BA.4/5 bivalent COVID-19 vaccines.

**Results:**

There were 299,967 children included in the analysis and 6,928 (2.3%) received one mRNA based 2023-2024 COVID-19 vaccine. Among all included children, 2,053 (0.7%) did not receive any doses prior to the 2023-2024 formulation; 605 (0.2%) received one prior dose, 1,834 (0.6%) received 2 prior doses, 2,236 (0.8%) received 3 prior doses and 200 (0.07%) received more than 3 prior doses.

**Conclusion:**

COVID-19 vaccine uptake in children aged 1-4 years was extremely low. A limited number of children received the 2023-2024 COVID-19 vaccine, and a larger proportion of 2023-2024 vaccinated children had not previously received the recommended multi-dose primary series. Novel approaches to improve vaccination coverage in this vulnerable population are warranted.

**Disclosures:**

Tara Ahi, MPH, Pfizer Inc.: Employee of Pfizer Inc. and may hold stock or stock options Jazmine S. Mateus, MPH, Pfizer Inc.: Employee of Genesis Research Group, which has received consulting fees from Pfizer Inc. Tiange Yu, MS, Pfizer Inc.: Employee of Genesis Research Group, which has received consulting fees from Pfizer Inc. Anan Zhou, MPH, Pfizer Inc.: Employee of Genesis Research Group, which has received consulting fees from Pfizer Inc. Rajeev M. Nepal, PhD, Pfizer Inc.: Employee of Pfizer Inc. and may hold stock or stock options Mary M. Moran, MD, Pfizer Inc.: Employee of Pfizer Inc. and may hold stock or stock options Alejandro Cané, MD, M.A., Pfizer Inc.: Employee of Pfizer Inc. and may hold stock or stock options Santiago M.C. Lopez, MD, Pfizer Inc.: Employee of Pfizer Inc. and may hold stock or stock options|Pfizer Inc.: Stocks/Bonds (Public Company) Laura A. Puzniak, PhD. MPH, Pfizer Inc.: Employee of Pfizer Inc. and may hold stock or stock options|Pfizer Inc.: Stocks/Bonds (Public Company) Kathleen M. Andersen, PhD, MSc, Pfizer Inc.: Employee of Pfizer Inc. and may hold stock or stock options

